# The selective 5-HT_1A_ receptor biased agonists, F15599 and F13714, show antidepressant-like properties after a single administration in the mouse model of unpredictable chronic mild stress

**DOI:** 10.1007/s00213-021-05849-0

**Published:** 2021-05-10

**Authors:** Monika Głuch-Lutwin, Kinga Sałaciak, Alicja Gawalska, Marek Jamrozik, Joanna Sniecikowska, Adrian Newman-Tancredi, Marcin Kołaczkowski, Karolina Pytka

**Affiliations:** 1 Department of Pharmacobiology, Faculty of Pharmacy, Jagiellonian University Medical College, Krakow, Poland; 2 Department of Pharmacodynamics, Faculty of Pharmacy, Jagiellonian University Medical College, Krakow, Poland; 3 Department of Medicinal Chemistry, Faculty of Pharmacy, Jagiellonian University Medical College, Krakow, Poland; 4 Neurolixis SAS, Castres, France

**Keywords:** Biased agonism, 5-HT_1A_ receptor, F15599, F13714, Depression

## Abstract

**Rationale:**

The prevalence of depression is ever-increasing throughout the population. However, available treatments are ineffective in around one-third of patients and there is a need for more effective and safer drugs.

**Objectives:**

The antidepressant-like and procognitive effects of the “biased agonists” F15599 (also known as NLX-101) which preferentially targets postsynaptic 5-HT_1A_ receptors and F13714, which targets 5-HT_1A_ autoreceptors, were investigated in mice.

**Methods:**

Antidepressant-like properties of the compounds and their effect on cognitive functions were assessed using the forced swim test (FST) and the novel object recognition (NOR), respectively. Next, we induced a depressive-like state by an unpredictable chronic mild stress (UCMS) procedure to test the compounds’ activity in the depression model, followed by measures of sucrose preference, FST, and locomotor activity. Levels of phosphorylated cyclic AMP response element-binding protein (p-CREB) and phosphorylated extracellular signal-regulated kinase (p-ERK1/2) were also determined.

**Results:**

F15599 reduced immobility time in the FST over a wider dose-range (2 to 16 mg/kg po) than F13714 (2 and 4 mg/kg po), suggesting accentuated antidepressant-like properties in mice. F15599 did not disrupt long-term memory consolidation in the NOR at any dose tested, while F13714 impaired memory formation, notably at higher doses (4–16 mg/kg). In UCMS mice, a single administration of F15599 and F13714 was sufficient to robustly normalize depressive-like behavior in the FST but did not rescue disrupted sucrose preference. Both F15599 and F13714 rescued cortical and hippocampal deficits in p-ERK1/2 levels of UCMS mice but did not influence the p-CREB levels.

**Conclusions:**

Our studies showed that 5-HT_1A_ receptor biased agonists such as F13714 and especially F15599, due to its less pronounced side effects, might have potential as fast-acting antidepressants.

**Supplementary Information:**

The online version contains supplementary material available at 10.1007/s00213-021-05849-0.

## Introduction

Depression is a severe mental condition, and according to the World Health Organization (WHO), more than 300 million people are affected by the disease globally, and by the end of 2030, they expect that depression will be the leading cause of disability worldwide (Lépine and Briley 2011). At present, the drugs available for the treatment of depression are still inadequate: around 60% of patients do not respond to antidepressants, and nearly one-third of them, despite receiving pharmacotherapy, experience relapse. Another limiting factor of most current antidepressant drugs is their delayed onset of action-clinical efficacy generally requiring several weeks of treatment. Delayed onset of action is a common cause of drug discontinuation, with about 28% of patients ceasing to take their antidepressant medications within 1 month, and 44% within 3 months of treatment (Lin et al. 1995). Alongside the typical depressive symptoms, such as continuous low mood, sadness, and feeling hopeless or helpless, patients often report anxiety or cognitive deficits (Gaspersz et al. 2018; Perini et al. 2019). As such, scientists are still searching for new compounds that have a fast onset of action, increased efficacy, and additional properties.

A possible way to achieve this objective is to develop novel serotonin 5-HT_1A_ receptor biased agonists. G-protein-coupled receptors (GPCRs) exist in many active conformations and coupled to various intracellular transducers (Wootten et al. 2018; Sałaciak and Pytka 2021). According to many studies, different ligands that bind to the same receptor may stabilize it in distinct conformations that preferentially activate specific signaling pathways, without affecting, or potentially even blocking, other transduction mechanisms (Śniecikowska et al. 2017; Wootten et al. 2018). This well-established paradigm is called functional selectivity or biased agonism. Compounds that possess such activity elicit distinct cellular responses, which may translate to different pharmacological and physiological outcomes. Biased agonists may, therefore, be fully selective and optimized drugs with increased efficacy and/or a reduced incidence of side effects.

The 5-HT_1A_ receptor is a GPCR which couples not only to the classical inhibitory G-protein regulated pathway but also to signaling pathways traditionally regulated by growth factors. This feature of the 5-HT_1A_ receptor enabled the development of functionally selective agonists at this target, the prototypical “biased agonists” being F15599 (also known as NLX-101) and F13714. F15599 preferentially activates postsynaptic 5-HT_1A_ heteroreceptors located in the prefrontal cortex or other brain regions, and F13714 is a preferential agonist of presynaptic autoreceptors in the raphe nuclei with more moderate postsynaptic activity (Newman-Tancredi 2011). In rats, F15599 stimulated extracellular signal-regulated kinase (ERK1/2) phosphorylation in the frontal cortex and inhibited this response in the hippocampus (Newman-Tancredi et al. 2009), which might be explained by preferential activation of Gαi versus other Gα subunits (Lin et al. 2002). Interestingly, both F15599 and F13714 possess antidepressant-and anxiolytic-like properties in naïve rats (Assié et al. 2010; Jastrzębska-Więsek et al. 2018). However, induction of serotonin syndrome, typified by flat body posture, forepaw treading, and lower lip retraction, was seen especially in the case of F13714 for which these effects were significantly more pronounced (Jastrzębska-Więsek et al. 2018). Recently, a rapid-onset antidepressant-like activity of F15599 was observed in rats subjected to unpredictable chronic mild stress (UCMS) procedure, a robust animal model of depression (Depoortère et al. 2019). Moreover, besides its mood-related properties, F15599 also improved animals’ cognitive performance in the novel object recognition test (Depoortère et al. 2019) and novel object pattern separation task (Van Goethem et al. 2015), and reversed the memory impairment caused by phencyclidine (Depoortère et al. 2010; Horiguchi and Meltzer 2012), unlike F13714. The broad scope of pharmacological properties of 5-HT_1A_ receptor biased agonists therefore encourages further investigation of their effects in diverse models and across different species.

Since the antidepressant-like and procognitive activity of F15599 and F13714 has never been tested in mice, in this study, we tested our hypothesis that the effects observed for both biased agonists also extend to this rodent species. In order to do that, we performed a battery of behavioral experiments in mice. We first evaluated the antidepressant-like and procognitive properties in the forced swim test (FST) and the novel object recognition test (NOR), respectively. Then, we used an UCMS model of depression and investigated whether the compounds show rapid antidepressant-like effect upon a single administration. As reference compounds, we used ketamine since it shows rapid antidepressant activity, and a selective serotonin reuptake inhibitor—fluoxetine (Krystal et al. 2019). We also evaluated if F15599 and F13714 influence the levels of phosphorylated cyclic AMP response element-binding protein (p-CREB) and phosphorylated ERK1/2 in the hippocampus and prefrontal cortex, two markers associated with antidepressant-like activity.

## Materials and methods

### Animals

In all experiments, we used naïve adult male Albino-Swiss CD-1 mice weighing 21 ± 2g (8 weeks old) and purchased from the Animal House at the Faculty of Pharmacy, Jagiellonian University Medical College, Kraków, Poland. Unless stated otherwise, the animals were kept in groups of 10 mice in standard cages (37 cm × 21 cm × 15 cm) at constant room conditions. Mice were used only once in each test. Behavioral experiments were performed between 8 a.m. and 4 p.m. and evaluated by a trained observer blind to the treatments. Mice were handled for 1 week before starting the experimental procedures. Animals were randomly allocated to the treatment using a computer-generated sequence and researchers making measurements on the animals or analyzing the results were blind to the allocation. Moreover, experimental groups were distributed across multiple cages and the location of the mouse cages in the room was changed following each day. After the end of behavioral procedures, mice were killed by cervical dislocation and the dissected tissues used for enzyme-linked immunosorbent assay (ELISA). All experimental procedures were approved by the Local Ethics Committee for Experiments on Animals in Kraków (approvals number 132/2017 and 159/2017), Poland, and performed under the guidelines provided by the European Union Directive of 22 September 2010 (2010/63/EU) and Polish legislation concerning animal experimentation.

### Drugs

3-Chloro-4-fluorofenylo-[4-fluoro-4-([(5-metylopirymidyn-2-ylo)-metyloamino]-metylo)piperydyn-1-ylo]-metanon (F15599) and 3-chloro-4-fluorofenylo-[4-fluoro-4-([(5-metylo-6-metyloamino-pirydyn-2-ylo)-metyloamino]-metylo)-piperydyn-1-ylo]-metanon (F13714) were synthesized in the Department of Medicinal Chemistry, Faculty of Pharmacy, Jagiellonian University. Both studied compounds, ketamine and fluoxetine (Sigma, Germany), were dissolved in water, and administered per os (po; F15599 and F13714) or intraperitoneally (ip; ketamine and fluoxetine) in a volume of 10 ml/ kg. Vehicle-treated groups received water. The doses of studied compounds for acute experiments were based on the studies performed in rats (Jastrzębska-Więsek et al. 2018). In the UCMS procedure, we chose for both compounds the doses for which the behavioral effect in the forced swim test was the most significant.

### Experiments in naïve mice

#### Forced swim test

Forced swim test was performed according to the method described by Porsolt and colleagues (Porsolt et al. 1977). Mice were placed individually for 6 min in glass cylinders (height 25 cm, diameter 10 cm) filled with water to a depth of 10 cm (23–25°C). Following a 2-min habituation period, total time spent immobile was recorded during the next 4 min. The animal was regarded as immobile when it remained floating passively in the water, making only small movements to keep its head above the water. The experiments were video-recorded and scored using aLab.io software by a trained observer blind to the treatments.

The experiments were performed 1 h after administration of the compounds.

#### Locomotor activity

The experiment was performed as previously described (Pytka et al. 2018). Locomotor activity was recorded individually for each mouse using activity cages made of transparent Perspex (40 cm × 40 cm × 31 cm, Activity Cage 7441, Ugo Basile, Italy). The cages were supplied with I.R. horizontal beam emitters connected to a counter for the recording of light-beam interruptions. Each mouse was placed in a cage for a 30-min habituation period. After that time, the number of crossings of photobeams was measured for 4 min (i.e., the time equal to the observation period in the forced swim test). The cages were disinfected with odorless veterinary disinfectant after each mouse.

The experiment was performed 1 h after compound administration.

#### Body temperature measurement

The experiment was performed as previously described (Pytka et al. 2015). The body temperature was measured using a Bioseb thermometer. The probe was introduced 20 mm into the rectus of the animal. First, we determined the base body temperature; i.e., we measured the rectal temperature three times and averaged the results. If the temperature varied by more than 0.6°C, the mouse was excluded from the study. Next, we administered the studied compound and measured body temperature after 30, 60, 90, and 120 min. The compound’s dose was divided by two until the disappearance of the effect and chosen based on the pilot studies.

In a separate experiment, we evaluated the effect of 5-HT_1A_ receptor antagonist, WAY 100635, on hypothermia induced by studied compounds (administered at the lowest hypothermic dose). After establishing the mean body temperature, we injected mice with WAY 100635 at the dose 0.3 mg/kg subcutaneously (*sc*) 15 min before the compound. The dose of WAY 100635 was based on our previous studies (Kubacka et al. 2016). Next, we measured the body temperature 30 and 60 min after compound administration.

The results were calculated as the body temperature change (Δ*t*):

$$ \Delta t=\mathrm{basal}\ \mathrm{body}\ \mathrm{temperature}-\mathrm{body}\ \mathrm{temperature}\ \mathrm{measured} $$

#### Novel object recognition

The test was performed according to the method described by Leger and colleagues (2013) and consisted of two sessions: familiarization and habituation. In the familiarization session, mice were placed individually in the open field (35 cm × 35 cm × 35 cm) with two identical objects (towers of Lego bricks or Falcon tissue culture flasks filled with sand) positioned 5 cm away from the walls. The animal’s head was positioned opposite the objects. Mice were left in the open field until they reached the 20-s criterion of total exploration, but no longer than 10 min. In the test phase, 24 h later, mice were placed again in the open field, but this time one of the objects was replaced with the new one (either a tower of Lego bricks or Falcon tissue culture flasks filled with sand). The position of the novel object (left or right) was randomized between each mouse and each group tested. Mice were again left in the open field until they reached the 20-s criterion of total exploration, but no longer than 10 min. After each test experiment, the objects and the open field were cleaned with odorless veterinary disinfectant to minimize any olfactory cues. The experiments were video-recorded and scored using aLab.io software by a trained observer blind to the treatments.

To evaluate the effect on memory consolidation, the compounds were administered after the familiarization phase.

### Unpredictable chronic mild stress in mice

The chronic mild stress model was originally established by Katz et al. (1981) and Katz (1982) and modified by Willner (1984). We performed the unpredictable chronic mild stress procedure according to the method described by Ruan et al. (2014). During the habituation period (1 week), mice were socially housed (4 per cage, normal housing conditions) to acclimate to the environment. For the unpredictable chronic mild stress procedure, we randomly divided 40 mice into five groups (8 mice per group). A control group (8 mice) was kept under standard housing conditions without disturbance except for required procedures, such as cage cleaning or behavioral testing. The rest of mice (4 groups of 8 mice) were subjected to unpredictable chronic mild stress procedure, i.e., housed singly and subjected daily to one out of ten stimuli in an unpredictable manner. The stimuli included (A) cold water swimming (13 ± 1°C, 5 min), (B) warm water swimming (37 ± 2°C, 5 min), (C) moist bedding (8 h), (D) cage tilt (45°, 8 h), (E) cage shaking (180 rpm, 10 min), (F) tail pinch (1 cm from the tip of the tail end, 1 min), (G) food deprivation (12 h), (H) water deprivation (12 h), (I) overnight illumination (12 h), or (J) no stress (24 h). The duration of the whole procedure was 4 weeks. After the end of the procedure, we administered the studied compounds once—60 min before the tests. We chose the doses of the studied compounds that caused the greatest reduction in the immobility in the forced swim test in naïve mice.

#### Sucrose preference test

The sucrose preference test was conducted according to the slightly modified method described by Filho et al. (2015). Before the proper test, mice were housed singly and given 72-h training to acclimate to the test procedures. In the first 24 h, access ad libitum to two feeding bottles of 1% (w/v) sucrose solution was given to each mouse. After 24 h, one bottle was replaced with tap water for the next 24 h. Then, mice were deprived of water and food for the third 24 h. On the test day, the weight of each bottle (1% sucrose solution and tap water) was recorded. Test animals were then given the bottles of 1% sucrose and water for 24 h. The consumed liquid weight was measured based on the weight of each bottle of fluids after the sucrose preference test minus the original starting weight. The percentage of sucrose solution intake was calculated using the following equation:

$$ \%\mathrm{sucrose}\ \mathrm{solution}\ \mathrm{intake}=\frac{\mathrm{sucrose}\ \mathrm{solution}\ \mathrm{intake}\ \left[\mathrm{g}\right]}{\mathrm{sucrose}\ \mathrm{solution}\ \mathrm{intake}\ \left[\mathrm{g}\right]+\mathrm{water}\ \mathrm{intake}\ \left[\mathrm{g}\right]}\times 100\% $$

### Ex vivo studies

#### p-CREB and p-ERK1/2 levels in the hippocampus and prefrontal cortex

After the behavioral assessments, mice were sacrificed, and their brains were rapidly removed and chilled in an ice-cold saline solution. The hippocampi were dissected on a cold plate, frozen, and stored at −80 °C until assay. On the day of experiments, tissues were thawed on ice and homogenized (1:9 w/v) in phosphate-buffered saline (4°C) and protease and phosphatase inhibitor cocktails were added. The 10% homogenates were prepared and homogenized for 30 s with TissueRuptor homogenizer. The homogenized tissues were centrifuged (2500×*g* at 4 °C for 20 min), and the supernatants were collected for further assays.

Protein concentrations of p-CREB and p-ERK1/2 in homogenates from hippocampi and prefrontal cortices were determined using the ELISA kits (SunRed) according to the manufacturer’s instructions. Serial dilutions of the standards were performed to make the standard curve within the range of this assay (p-CREB: 0.5–72 ng/ml; p-ERK1/2: 10–3000 pg/ml). The samples were analyzed in duplicates, and the mean concentrations were calculated. p-CREB and p-ERK1/2 antibodies are high selectivity and thus did not cross-react with any other cytokines. The reaction was terminated after the stop solution was added. The intensity of the color was read at 450 nm. Absorbance was measured in a multifunction plate reader (POLARstar Omega, BMG Labtech, Germany).

### Statistical analysis

The number of animals in groups was based on our previous experiments (Pytka et al. 2017). Results are presented as box plots or means ± SEM. The normality of data sets and their homogeneity were determined using Shapiro-Wilk and Brown-Forsythe test, respectively. Except for the memory performance and body temperature measurement, comparisons between experimental and control groups were performed by one-way ANOVA, followed by Newman-Keuls post hoc. In the body temperature, measurement effects of treatments were compared using a two-way ANOVA with treatment as a between-animal variable, and time as a within-animal variable, followed by Newman-Keuls post hoc. The reported *p* values of all ANOVAs used the Geisser-Greenhouse correction when the sphericity assumption was not met. To assess memory performances, the mean times the mice spent exploring each object during test session are compared with the chance level (10 s, equal exploration of the objects) through a univariate *t* test.

## Results

### Experiments in naïve mice

#### Forced swim test

The data sets for the experiments showed normal distribution as well as homogeneity of the variance (F15599 (*F*(5,54) = 1.826, ns) and F13714 (*F*(5,57) = 2.375, ns)).

**Fig. 1.**
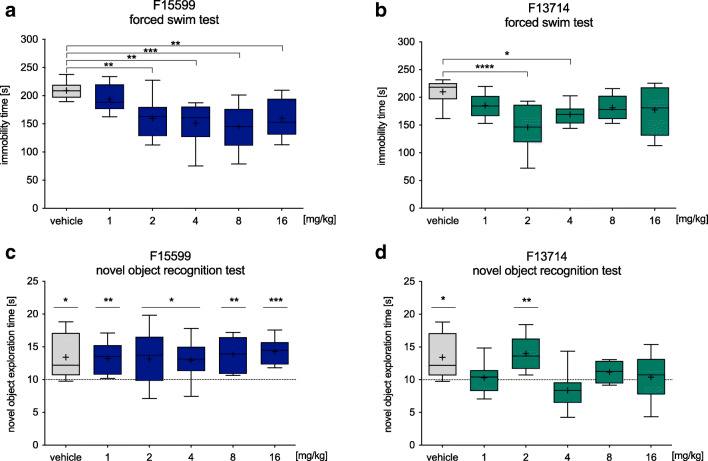
The effect of F15599 (panel **a**) and F13714 (panel **b**) on mice behavior in forced swim test and the novel object recognition test (panels **c**, **d**). All the tested compounds or water were administered 60 min (per os) before the test. Statistical analysis: Shapiro-Wilk test for normality; forced swim test: Brown-Forsythe test for homogeneity of variance and one-way ANOVA (Newman-Keuls post hoc); novel object recognition test: one-sample *t*-test (chance level = 10 s) *p < 0.05, **p < 0.01, ***p<0.001, ****p < 0.0001; *n* = 8–11 mice per group

F15599 at the doses 2, 4, 8, and 16 mg/kg (but not 1 mg/kg) decreased immobility by 23.5%, 27.6%, 30.6%, and 23.3%, respectively, compared with control animals (*F*(5,54) = 6.586, *p* < 0.0001; Fig. 1a). F13714 decreased the immobility in mice at the doses 2 and 4 mg/kg by 30.3% and 19.5% (*F*(5,57) = 5.719, *p* < 0.001; Fig. 1b).

#### Locomotor activity

The data sets for the experiments showed normal distribution as well as homogeneity of the variance (F15599 (*F*(4,35) = 1.355, ns) and F13714 (*F*(2,21) = 1.605, ns)).

F15599 at the dose 16 mg/kg (but not 2, 4 or 8 mg/kg) decreased locomotor activity by 70.0% compared with vehicle-treated mice (*F*(4,35) = 2.888, *p* < 0.05; Table 1). F13714 did not influence locomotor activity of mice at any of the tested doses (*F*(2,21) = 3.231, ns; Table 1).

**Table 1:** The influence of F15599 and F13714 on locomotor activity in mice

Treatment	Dose (mg/kg)	Number of crossings ± SEM
Vehicle	-	184.3 ± 30.7
F15599	2	146.1 ± 40.9
	4	129.5 ± 19.9
	8	118.6 ± 15.2
	16	55.3 ± 17.2**
Vehicle	-	183.0 ± 24.1
F13714	2	125.4 ± 12.3
	4	124.3 ± 19.9

The locomotor activity was recorded individually for each animal in activity cages. After habituation time (30 min), the number of crossings of photobeams was measured during next 4 min. The tested compounds or water (vehicle) were administered per os 60 min before the test. Statistical analysis: Shapiro-Wilk test for normality; Brown-Forsythe test for homogeneity of variance and one-way ANOVA (Newman-Keuls post hoc); **p < 0.01, *n*=8–10 mice per group

#### Novel object recognition

The data sets for the experiments with F15599 and F13714 showed normal distribution.

Following a single administration of F15599 at all doses, the mean times the mice spent exploring the novel object during test session significantly differed from the chance level of 10 s (Fig. 1c). This indicates that F15599 did not interfere with the increased exploration of the novel object. In the case of F13714, the mean novel object exploration time was significantly different from the chance level only at the dose of 2 mg/kg. In contrast, the other doses (1, 4, 8, or 16 mg/kg; Fig. 1d) were not significantly different from chance level, suggesting that F13714 had impacted memory retention.

#### The influence on body temperature

All data sets for the experiments showed normal distribution.

F15599 (4–16 mg/kg) and F13714 (0.5–2 mg/kg) given alone significantly and dose-dependently decreased rectal body temperature in mice during a 2-h measurement.

**Fig. 2:**
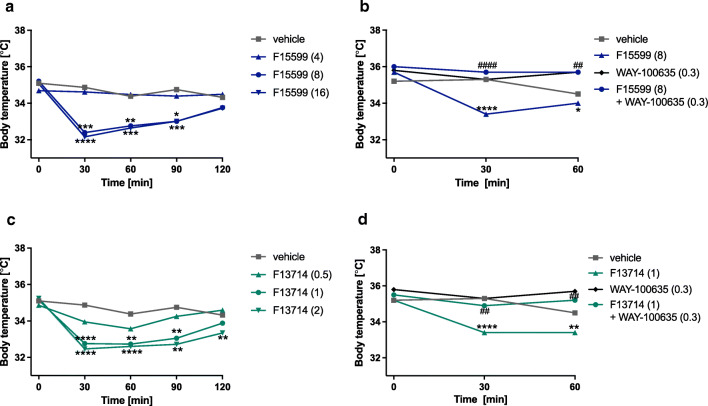
The effect of the administration of F15599 (panels **a**, **b**) or F13714 (panels **c**, **d**) alone and in combination with WAY-100635 on body temperature in mice. After measuring the basal temperature of mice, the tested compounds or water (vehicle) were administered per os. The temperature was measured after 30, 60, 90, and 120 min after compound administration. Next, again after measuring the basal body temperature of mice, WAY-100635 was injected at the dose 0.3 mg/kg subcutaneously, and after 15 min, the tested compounds or water (vehicle) were administered per os. The temperature was measured after 30 and 60 min after compound administration. The doses of compounds are in brackets. Statistical analysis: Shapiro-Wilk test for normality, and two-way ANOVA (Newman-Keuls post hoc); *p<0.5, **p<0.01, ***p<0.001, ****p<0.0001 vs. vehicle-treated group, #*p*<0.05, ##*p*<0.01, ###*p*<0.001 vs. group treated with a compound alone; *n*=7-10 mice per group

F15599 at doses 8 and 16 mg/kg significantly decreased the temperature after 30, 60, and 90 min (treatment: *F*(3,36)=13.15, *p*<0.0001; time × treatment: *F*(9,108)=5.067, *p*<0.0001) (Fig. 2a). None of tested doses induced hypothermia after 120 min post injection. The decrease in body temperature induced by lower dose of F15599 was significantly attenuated by WAY-100635 (0.3 mg/kg) after 30 and 60 min post injection (treatment: *F*(3,33)=12.64, *p*<0.0001; time × treatment: *F*(3,26)=5.123, *p*<0.01) (Fig. 2b).

F13714 at doses 1 and 2 mg/kg significantly decreased the temperature after 30, 60, and 90 min (treatment: *F*(3,36)=15.68, *p*<0.0001; time × treatment: *F*(9,108)=2.556, *p*<0.05) (Fig. 2c). After 2 h only, the dose 2 mg/kg significantly induced hypothermia in mice. The decrease in body temperature induced by lower dose of F13714 was significantly abolished by WAY-100635 (0.3 mg/kg) after 30 and 60 (treatment: *F*(3,29)=12.76, *p*<0.0001; time × treatment: *F*(3,27)=3.354, *p*<0.05) post injection (Fig. 2d).

### Unpredictable chronic mild stress in mice

#### Forced swim test

The data sets for the experiments showed normal distribution as well as homogeneity of the variance (data sets for F15599, F13714, and ketamine: *F*(4,37) = 2.110, ns; data sets for fluoxetine: *F*(2,21)=1.309, ns).

**Fig. 3:**
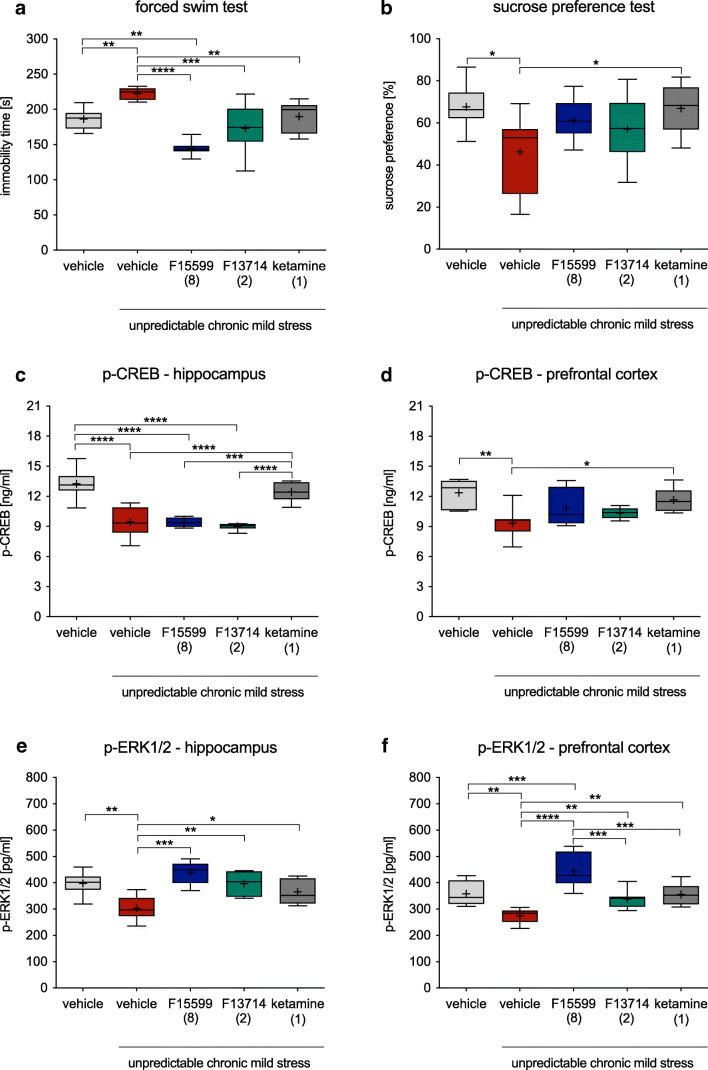
The effect of F15599, F13714 and ketamine on the: immobility in the forced swim test (panel **a**), sucrose preference (panel **b**), as well as p-CREB and p-ERK 1/2 levels in the hippocampus (panels **c**, **e**), and the prefrontal cortex (panels **d**, **f**) in mice under the unpredictable chronic mild stress procedure. Unpredictable chronic mild stress procedure was performed for 4 weeks. Twenty-four hours after last stressor, behavioral tests were performed. All the tested compounds or water (vehicle) were administered 60 min (per os) before the test. After behavioral testing, mice were killed and prefrontal cortices and hippocampi were collected for further research. The doses of compounds are in brackets. Statistical analysis: Shapiro-Wilk test for normality, Brown-Forsythe test for homogeneity of variance, and one-way ANOVA (Newman-Keuls post hoc *p < 0.05, **p < 0.01, ***p<0.001, ****p < 0.0001; *n* = 8–10 mice per group

Mice subjected to the UCMS procedure, compared with non-stressed animals, showed significantly increased immobility (by 19.5%) in the FST compared with non-stressed animals (*F*(4,37) = 14.01, *p* < 0.0001; Fig. 3a). A single injection of either F15599 (8 mg/kg) or F13714 (2 mg/kg), or the reference drug, ketamine (1 mg/kg), reversed the stress-induced increase in the immobility in mice (Fig. 3a). It was noted that F15599 did not just reverse the effects of the UCMS, but actually reduced mean immobility time to 22.4% below that of the non-stressed control (Fig. 3a). However, a single injection of fluoxetine did not attenuate the decreased immobility in the stressed mice (*F*(2,21)=12.89, *p*<0.001; Figure S1A).

#### Sucrose preference test

The data sets for the experiments showed normal distribution as well as homogeneity of the variance (data sets for F15599, F13714 and ketamine: *F*(4,35) = 0.6841, ns; data sets for fluoxetine: *F*(2,21)=0.2567, ns).

Stressed mice, compared with non-stressed controls, showed significantly decreased by 31.6% preference for 1% sucrose solution (*F*(4,35) = 3.335, *p* < 0.05; Fig. 3b). A single injection of the reference drug, ketamine, but not F15599 or F13714, reversed the stress-induced decrease in 1% sucrose solution preference (Fig. 3b). Similarly, an acute fluoxetine administration was not enough to attenuate the decreased sucrose preference (*F*(2,21)=38.84, *p*<0.0001; Figure S1B).

#### Locomotor activity

The data sets for the experiments showed normal distribution as well as homogeneity of the variance (data sets for F15599, F13714 and ketamine: *F*(4,35) = 0.8717, ns; data sets for fluoxetine: *F*(2,21=0.0802, ns).

None of the treatments influenced the number of crossings of photobeams over the 4-min testing period in mice (data sets for F15599, F13714, and ketamine: *F*(4,35) = 0.9541, ns—Table 2; data sets for fluoxetine: *F*(2,21) = 0.7968, ns—Table S1).

**Table 2:** The influence of F15599, F13714, and ketamine on locomotor activity in mice under the unpredictable chronic mild stress procedure

Treatment	UCMS	Dose (mg/kg)	Number of crossings ± SEM
Vehicle	No	-	452.0 ± 50.5
Vehicle	Yes	-	461.3 ± 58.8
F15599	Yes	8	483.5 ± 52.1
F13714	Yes	2	411.3 ± 46.8
Ketamine	Yes	1	538.8 ± 43.8

After 4 weeks of unpredictable chronic mild stress procedure, the locomotor activity was recorded individually for each animal in activity cages. After habituation time (30 min), the number of crossings of photobeams was measured during next 4 min. The tested compounds or water (vehicle) were administered per os 60 min before the test. Ketamine was administered intraperitoneally 30 min before the test. Statistical analysis: one-way ANOVA (Newman-Keuls post hoc); *n*=8-10 mice per group

#### p-CREB and p-ERK1/2 levels in the hippocampus and prefrontal cortex

The data sets for the experiments assessing the p-CREB (hippocampus: *F*(4,25) = 1.676, ns; prefrontal cortex: *F*(4,30) = 1.233, ns) and p-ERK1/2 levels (hippocampus: *F*(4,23) = 0.1493, ns; prefrontal cortex: *F*(4,36) = 1.061, ns) showed normal distribution as well as homogeneity of the variance.

Stressed mice showed significant decrease in p-CREB level in the hippocampus (*F*(4,25) = 20.40, *p* < 0.0001; Fig. 3c) and the prefrontal cortex (*F*(4,30) = 4.661, *p* < 0.01; Fig. 3d). A single injection of the reference drug, ketamine, but not F15599 or F13714, significantly reversed the stress-induced decrease of p-CREB level in the hippocampus and the prefrontal cortex (Fig. 3).

The level of p-ERK1/2 for stressed mice was significantly lower in the hippocampus (*F*(4,23) = 6.986, *p* < 0.001; Fig. 3e) and the prefrontal cortex (*F*(4,36) = 16.34, *p*<0.0001; Fig. 3f). A single injection of the reference drug ketamine or of F15599 or F13714 reversed the stress-induced decrease of p-ERK1/2 level in the hippocampus and the prefrontal cortex (Fig. 3). Furthermore, F15599, compared with non-stressed controls, significantly increased p-ERK1/2 level in the prefrontal cortex (Fig. 3).

## Discussion

The key findings of the present study are as follows: (i) the 5-HT_1A_ receptor “biased agonists,” F15599 and F13714, demonstrated antidepressant-like activity in the FST in mice; however, F15599 was effective over a broader dose range than F13714. (ii) F15599 presented more favorable properties in the NOR test than F13714. Thus, whereas F15599, given at antidepressant-like doses, did not interfere with cognitive function in this test, F13714 impaired long-term memory consolidation. (iii) In mice subjected to UCMS, a single administration of each compound reversed depressive-like behaviors in the forced swim test but was not enough to ameliorate anhedonia-like behavior, as assessed by sucrose intake. (iv) F13714 potently elicited hypothermia in mice, a marker of pre-synaptic 5-HT_1A_ receptor activation in this species, whereas F15599 was less potent. Finally, (v) on a cellular level, a single dose of F15599 and F13714 up-regulated p-ERK1/2 levels in both hippocampus and prefrontal cortex in the stressed mice.

Multiple 5-HT_1A_ receptor subpopulations are expressed in different brain regions, where they couple to distinct cellular signaling pathways and mediate various physiological responses, which may be desirable therapeutic effects or undesirable side-effects (reviewed in Chilmonczyk et al. 2015; Sałaciak and Pytka 2021). The discovery that the 5-HT_1A_ receptor couples to different signaling pathways permitted the development of “biased agonists,” such as F15599 and F13714, which are able to differentiate between physiological effects and which were the subject of our current study.

First, we showed that F15599 and F13714 produced antidepressant-like effects in the forced swim test. Since neither compound increased the locomotor activity of animals (Table 1), the observed effects can be considered to be specifically due to an influence on despair-like behaviors. Our results differ from those obtained by Jastrzębska-Więsek and colleagues (2018) or those of Assié et al. (2010) in rats. The minimum effective dose (MED) for immobility reduction in mice was over 3-fold higher than the MED for antidepressant-like effects in rats. Moreover, in rats, immobility duration at 2.5 (F13714) or 5 mg/kg (F15599) was reduced almost to 0, whereas in mice, the greatest reduction of immobility was around 31% at 2 (F13714) or 8 mg/kg (F15599). Interestingly, while F15599 decreased the immobility time in mice over a wide range of doses (i.e., 2–16 mg/kg), F13714 showed an inverted U-shaped dose-effect (only two doses were active, i.e., 2 and 4 mg/kg). This non-linear dose-effect relationship is prevalent in pharmacological studies but very poorly understood (Baldi and Bucherelli 2005). There are several possible explanations for this phenomenon. First, F13714 might have behavioral effects in mice which manifest themselves at higher doses and interfere with its anti-immobility properties in the FST. However, in the case of F15599, there was a decrease in locomotor activity at the highest dose tested and this did not prevent its anti-immobility effects (Fig. 1). A more likely explanation, therefore, is the regional selectivity of the compounds. In ^18^F-FDG PET studies, F15599 induced a clear increase in labeling (reflecting increased activation) in the frontal cortex (Levigoureux et al. 2019), while F13714 increased blood oxygen dependence in the cingulate and motor cortex, striatum, or thalamus (Becker et al. 2016). Given that 5-HT_1A_ receptors in the frontal cortex are crucial for antidepressant-like effect, this might be the main reason why F15599 (which shows biased agonism for 5-HT_1A_ receptors in this brain region) has more prominent activity than F13714 for reduction of immobility in the forced swim test.

To complement the interpretation of the forced swim test results, we determined the influence of the biased agonists on body temperature in mice. It is well established that the hypothermic effect of 5-HT_1A_ agonists in mice is mediated by presynaptic 5-HT_1A_ receptors (Goodwin et al. 1985). Here, both F15599 and F13714 caused a reduction in body temperature in mice, but F13714 was 8-fold more potent than F15599 (based on MED ratios). For both agonists, the hypothermic effect was reversed by WAY-100635, a selective 5-HT_1A_ receptor antagonist. The present results are in agreement with these findings; i.e., F13714 administered even at a low dose (1 mg/kg) activated presynaptic 5-HT_1A_ receptors (as shown by hypothermia induction), whereas low doses of F15599 did not affect these receptors. However, at higher doses, the selectivity of F15599 for postsynaptic receptors gradually disappeared, as shown by the fact that it elicited hypothermia at 8 and 16 mg/kg.

As well as regulating mood, 5-HT_1A_ receptors influence multiple other responses via the activity of glutamatergic, cholinergic, and possibly GABAergic neurons in the cerebral cortex, hippocampus, and the septohippocampal projection, thereby affecting also memory functions. Data on the effects of 5-HT_1A_ agonists on memory processes are ambiguous (reviewed in Glikmann-Johnston et al. 2015).

Here, we showed that F15599 did not impair memory formation in the novel object recognition test at any of the doses tested, while F13714 impaired memory consolidation at 4 of the 5 doses tested (except 2 mg/kg). Our results are in agreement with the previous studies on 5-HT_1A_ biased agonists. Van Goethem et al. (2015) showed that the activation of postsynaptic 5-HT_1A_ heteroreceptors by F15599 improved pattern separation in a spatial memory test. In contrast, F13714 impaired performance in this test, presumably due to its preferential stimulation of raphe-located autoreceptors. Another explanation of memory impairment may be related to the activation of specific signaling pathways. We know that cognitive deficits are linked to adenyl cyclase inhibition, while the memory-enhancing effect involves increased ERK1/2 phosphorylation. F15599 increases the level of p-ERK1/2 to a greater extent than F13714 (Newman-Tancredi 2011) and, therefore, we did not observe the deteriorating effect of F15599 on memory consolidation. It is noteworthy that F13714 showed an inverted U-shaped dose-effect, similar to that seen in the forced swim test. Since memory formation is a multifactorial process, understanding this non-linear dose-response effect requires more experiments.

Finally, we evaluated the antidepressant-like effect of F15599 and F13714 using the UCMS model of depression in mice. This model is considered among the best animal models of depression, reproducing not only behavioral (anhedonia) but also neurobiological aspects of the human pathology and also exhibiting a high degree of validity and translational potential (Willner 2017). For the present experiment, we chose the doses of the studied compounds that caused the greatest reduction in the immobility in the FST in naïve mice. We showed that a single administration of each “biased agonist” was sufficient to induce an antidepressant-like effect in the FST in the mice subjected to UCMS. Moreover, this effect caused by biased agonists was stronger than that observed for ketamine. In contrast, a single injection with fluoxetine was not enough to reverse changes observed in the FST and sucrose preference test (SPT). The agonists did not, however, reverse the anhedonic-like deficit in the sucrose preference test in the stressed mice, unlike ketamine. As a comparison, a recent study in rats by Depoortère et al. (2019) showed that F15599, administered *ip* twice daily, reversed the chronic mild stress-induced decrease in sucrose intake. This was observed from day 1 of testing and was maintained throughout the 2 weeks of treatment and up to 4 weeks after cessation of treatment. It is unclear why a reversal of sucrose preference was not seen in our experiment. The possible explanations include the different routes of administration and/or dosing, as well as species. In our study, we administered the compounds acutely once po, while in the study by Depoortère and colleagues, F15599 was injected *ip* twice a day. Moreover, studies show that the anti-anhedonic rapid action of ketamine is not always obtained, and it does not depend on under-dosing of ketamine (Papp et al., 2017; Willner et al., 2019). These inconsistent results imply that even the choice of strain and experimental conditions influence the onset of ketamine response (Li et al., 2011; Papp et al., 2017; Ramaker and Dulawa, 2017; Willner et al., 2019). Similar findings are observed in mice—the efficacy of ketamine may be related to the choice of stress conditions, the strain of mice, or different protocol of sucrose preference test (Krzystyniak et al., 2019; Ma et al., 2013; Tan et al., 2017; Tian et al., 2018; Xiong et al., 2018). Nevertheless, the present study provides the first evidence that F15599 induces a very rapid antidepressant-like response in mice and supports the assertion that preferential biased agonist targeting of postsynaptic 5-HT_1A_ receptors is a promising strategy for improved treatment of mood deficits.

The unpredictable chronic mild stressed procedure caused a decrease in the level of p-CREB and p-ERK1/2 in the hippocampus and prefrontal cortex. Many studies support the crucial role of ERK1/2 and its downstream transcription factor, CREB, in the pathogenesis, symptomatology, and treatment of depression (Blendy 2006; Wang and Mao 2019). In both humans and chronic animal models of depression, ERK signaling was significantly downregulated in the prefrontal cortex and hippocampus, two core areas implicated in depression (Dwivedi et al. 2001, 2006; First et al. 2011). Here, both 5-HT_1A_ receptor biased agonists reversed p-ERK1/2 deficits, and neither influenced p-CREB levels in either brain structure. Interestingly, the effect of F15599 in the prefrontal cortex was significantly more pronounced than that of F13714 or even ketamine. Altered expression of ERK1/2 has been shown in both human and animal studies in the context of depressive states. In human *postmortem* studies, scientists showed a decreased ERK1/2 activity and expression in the prefrontal cortex and hippocampus, two major brain structures affected by depression (Dwivedi et al. 2001; Liu et al. 2017). Animal studies confirm these findings, and drugs such as fluoxetine or ketamine normalize ERK1/2 levels in depression models (Qi et al. 2008; First et al. 2011; Li et al. 2010; Zhou et al. 2014; Miller et al. 2014; Ma et al. 2017; Lee et al. 2019). Thus, a rapid increase in ERK1/2 phosphorylation may be a critical factor of a rapid antidepressant response and the present findings therefore suggest that the rapid and robust antidepressant-like properties of F15599 in mice are mediated by activation of this intracellular response.

Our study has some limitations. First, we did not assess the tested compounds’ activity in the novel object recognition test after the UCMS, as we wanted to investigate and highlight the antidepressant-like rather than procognitive effects in the depression model. The experiments on naïve animals were performed to eliminate the possibility of a 5-HT_1A_ receptor-mediated deteriorating effect on memory. Moreover, we administered the compounds acutely as we intended to show similarities between the studied biased 5-HT_1A_ agonists and rapid-acting ketamine. However, considering that neither F15599 nor F13714 reversed the stressed mice’s anhedonic-like behavior in the SPT and based on studies in rats (Depoortère et al. 2019), we may speculate that the compounds need to be administered at least twice or chronically. This issue will require future studies. Besides, we only investigated compounds’ influence on p-CREB and p-ERK1/2 levels in the stressed animals. However, how the compounds influence these signaling molecules in naïve mice remains an open question. Moreover, in our experiments, we did not record basal locomotor activity, as we intended to prove that the overall locomotor activity of groups that received the compounds did not differ significantly from controls, which received only the vehicle.

## Conclusion

The present study shows, for the first time, that F15599 and F13714 possess antidepressant-like activity in the FST in naïve mice. However, F15599 produced antidepressant-like effects over a broader dose range and showed more favorable properties in a test assessing cognitive function; i.e., it did not impair long-term memory consolidation, whereas F13714 did. In a UCMS model, a single administration of each compound reversed depressive-like behaviors in the forced swim test but did not ameliorate anhedonia. In addition, at a cellular level, a single dose of F15599 elicited very robust up-regulation of p-ERK1/2 levels in both hippocampus and prefrontal cortex in the stressed mice, whilst F13714 exerted a less marked effect. Overall, the present observations suggest that functionally selective 5-HT_1A_ agonists that rapidly increase ERK1/2 phosphorylation levels may have potential as fast-acting antidepressants.

## Supplementary Information

ESM 1 (DOCX 99 kb)
