# Supplementary material for: The selective 5-HT1A receptor biased agonists, F15599 and F13714, show antidepressant-like properties after a single administration in the mouse model of unpredictable chronic mild stress
Source: Psychopharmacology (Berl). 2021 May 10;238(8):2249–60. doi: 10.1007/s00213-021-05849-0 (PMC8292235; doi:10.1007/s00213-021-05849-0)
Supplement: Supplementary file 1 — (DOCX 99 kb) [file 213_2021_5849_MOESM1_ESM.docx]

**Table S1.** **The influence of fluoxetine on locomotor activity in mice under the unpredictable chronic mild stress procedure.**

| **Treatment** | **UCMS** | **Dose (mg/kg)** | **Number of crossings ± SEM** | | |
| --- | --- | --- | --- | --- | --- |
| vehicle | no | - | 439.4 | ± | 70.6 |
| vehicle | yes | - | 404.5 | ± | 67.1 |
| fluoxetine | yes | 10 | 516.5 | ± | 52.7 |

After 4 weeks of unpredictable chronic mild stress procedure the locomotor activity was recorded individually for each animal in activity cages. After habituation time (30 min) the number of crossings of photobeams was measured during next 4 min. Fluoxetine was administered intraperitoneally 30 min, and vehicle *per os* 60 min before the test. Statistical analysis: Shapiro-Wilk test for normality; Brown-Forsythe test for homogeneity of variance and one-way ANOVA (Newman-Keuls *post hoc*); n=8 mice per group.

**Figure S1. The effect of fluoxetine on the: immobility in the forced swim test (Panel A) and the sucrose preference (Panel B) in mice under the unpredictable chronic mild stress procedure.** Unpredictable chronic mild stress procedure was performed for 4 weeks. 24h after last stressor, behavioral tests were performed. Fluoxetine was administered intraperitoneally 30 min, and vehicle *per os* 60 min before the test. The dose of compound is in brackets. Statistical analysis: Shapiro-Wilk test for normality, Brown-Forsythe test for homogeneity of variance, and one-way ANOVA (Newman-Keuls *post hoc* ∗∗∗p < 0.001, ∗∗∗∗p < 0.0001); n = 8 mice per group.
